# Deciphering the mode of action of a mutant *Allium sativum* Leaf Agglutinin (mASAL), a potent antifungal protein on *Rhizoctonia solani*

**DOI:** 10.1186/s12866-015-0549-7

**Published:** 2015-10-26

**Authors:** Prithwi Ghosh, Amit Roy, Daniel Hess, Anupama Ghosh, Sampa Das

**Affiliations:** Division of Plant Biology, Bose Institute, Centenary Campus, P1/12, CIT Scheme, VIIM, Kankurgachi, Kolkata, 700054 West Bengal India; The Protein Analysis Facility, Friedrich Miescher Institute for Biomedical Research, Basel, Switzerland; Present address: Chemical Ecology, Department of Plant Protection Biology, Swedish University of Agricultural Sciences, Alnarp, SE-230 53 Sweden

**Keywords:** Mutant *Allium sativum* leaf agglutinin, *Rhizoctonia solani*, Antifungal proteins, Molecular targets, PCD, Plasma membrane permeabilization, LC-MS/MS

## Abstract

**Background:**

Mutant *Allium sativum* leaf agglutinin (mASAL) is a potent, biosafe, antifungal protein that exhibits fungicidal activity against different phytopathogenic fungi, including *Rhizoctonia solani*.

**Methods:**

The effect of mASAL on the morphology of *R.solani* was monitored primarily by scanning electron and light microscopic techniques. Besides different fluorescent probes were used for monitoring various intracellular changes associated with mASAL treatment like change in mitochondrial membrane potential (MMP), intracellular accumulation of reactive oxygen species (ROS) and induction of programmed cell death (PCD). In addition ligand blot followed by LC-MS/MS analyses were performed to detect the putative interactors of mASAL.

**Results:**

Knowledge on the mode of function for any new protein is a prerequisite for its biotechnological application. Detailed morphological analysis of mASAL treated *R. solani* hyphae using different microscopic techniques revealed a detrimental effect of mASAL on both the cell wall and the plasma membrane. Moreover, exposure to mASAL caused the loss of mitochondrial membrane potential (MMP) and the subsequent intracellular accumulation of reactive oxygen species (ROS) in the target organism. In conjunction with this observation, evidence of the induction of programmed cell death (PCD) was also noted in the mASAL treated *R. solani* hyphae.

Furthermore, we investigated its interacting partners from *R. solani*. Using ligand blots followed by liquid chromatography tandem mass spectrometry (LC-MS/MS) analyses, we identified different binding partners including Actin, HSP70, ATPase and 14-3-3 protein.

**Conclusions:**

Taken together, the present study provides insight into the probable mode of action of the antifungal protein, mASAL on *R. solani* which could be exploited in future biotechnological applications.

**Electronic supplementary material:**

The online version of this article (doi:10.1186/s12866-015-0549-7) contains supplementary material, which is available to authorized users.

## Background

After blast disease, sheath blight is the most devastating disease of rice [1]. Sheath blight is caused by a soil-borne, necrotrophic, basidomycete fungal pathogen, *Rhizoctonia solani* Kühn (teleomorph *Thanatephorus cucumeris* anastomosis group 1-IA). The disease affects 15–20 million ha of rice fields and causes a yield loss of 6 million tons of rice grain per year in Eastern Asia [2]. Management of rice sheath blight is difficult due to the wide host range of the pathogen, its high genetic variability and its ability to survive in soil for a long period of time and also because of the non-availability of genetic resistance among rice cultivars [3]. Consequently, the only widely used method to effectively control the disease is the use of chemical fungicides. However, one of the major limitations of this practice is its harmful effect on public health and environment [4]. In addition, the development of fungicidal resistance is an emerging problem in the protection of plants against fungi, making the task of controlling fungal pathogens more challenging [5, 6]. Due to these limitations, genetic manipulation of crop plants to induce expression of antifungal proteins is emerging as an attractive method to control fungal pathogens. These antifungal proteins are produced by wide range of organisms, including humans, amphibians, arthropods, plants, and fungi [7–9]. They act on diverse cellular targets and exhibit different modes of action. For instance, some antifungal peptides target cell wall and interfere with membrane permeability. Others are reported to undergo receptor-mediated internalization, followed by production of reactive oxygen species (ROS) and induction of apoptosis [10, 11]. Several studies carried out during the past few decades have shown that transgenic crop plants expressing different antifungal proteins exhibit increased resistance to fungal pathogens with no adverse effects on plant metabolism or crop yield [12, 13]. Mannose-binding monocot lectins belong to one such group of proteins that are inherently capable of protecting plants and organisms from diverse predators and pathogens [14, 15]. The biological roles of lectins in protecting crop plants vary considerably and depend upon their oligomerization status [16]. For instance dimeric lectins are insecticidal, monomeric ones are fungicidal [17] and tetramers exhibit anti-retroviral properties [18]. Our group has developed a novel and biosafe [19] monomeric antifungal protein called mASAL by introducing five site-specific mutations in the potent homodimeric insecticidal lectin *Allium sativum* leaf agglutinin (ASAL). This newly developed 12-kDa protein displayed fungicidal activity against several phytopathogenic fungi namely, *Rhizoctonia solani, Fusarium oxysporum,* and *Alternaria brassicicola* [20]. Because of its potent antifungal activity, mASAL poses itself to be used in agricultural biotechnology to combat fungal diseases. However, to fully exploit the potential of mASAL as an antifungal agent, a detailed understanding of its mode of action is absolutely necessary. A previous study from our group revealed the intracellular localization of the protein when fungal cells were treated with mASAL [20]. The small molecular size of mASAL, favors in penetrating through fungal cell walls, since the size exclusion limit for a typical antifungal protein ranges between 15 and 20 kDa [21].

The present study aimed at getting additional detailed insights in to the mechanism of action of mASAL. We investigated its putative interacting partners within *R. solani* cells. This is the first report on the identification of putative interaction partners of mASAL from *R. solani*. Besides, we found that exposure to mASAL leads to morphological anomalies, change in membrane permeability, ROS generation and DNA fragmentation. Taken together the data obtained through this study provide a glimpse of possible underlying mechanisms that have been employed by mASAL to exert its antifungal activity.

## Methods

### Fungal strains and growth conditions

*R. solani* (MTCC code-4633) used for the experiments was obtained from IMTEC, Chandigarh, India. The cultures were either maintained aseptically on potato dextrose agar (PDA) in 90 mm plate or in potato dextrose broth (PDB) at 28 °C in darkness. Liquid cultures were maintained by inoculating 50 ml of PDB medium in a 250 ml Erlenmeyer flask with a piece of fresh mycelia from PDA, with agitation at 180 rpm for 3 days.

### Expression and purification of mASAL

Mutant *Allium sativum* leaf agglutinin (mASAL) was expressed and purified according to the previously described protocol [19]. Breifly, mASAL was cloned in pET28a + vector and transformed into *E. coli* BL21 cell line (Invitrogen, CA, USA). 500 ml of Luria broth (LB) medium was inoculated with 10 ml of overnight grown bacterial culture. The culture was then grown at 37 °C with shaking at 180 rpm until an optical density of 0.5 to 0.8 at 600 nm was reached. The recombinant protein was expressed following induction with 0.3 mM Isopropyl β-D-thiogalactopyranoside (IPTG) and incubated with constant shaking at 180 rpm for 16 h at 16 °C. Purification was carried out following manufacturers’ instructions with some modification (Qiaexpressionist, Qiagen, Germany). His-tagged proteins were purified by metal-affinity chromatography using Ni-NTA column [19].

### Microscopy

The effect of mASAL on the hyphal morphology of *R. solani* was observed using optical microscopy, scanning electron microscopy (SEM) and fluorescence microscopy. For sample preparation *R. solani* cells were cultured for three days at 28 °C followed by incubation with 20 μg/ml mASAL for 24 h. As a control, the cells were treated with similar volumes of PBS for the same time period. *R. solani* mycelia were also stained with different fluorescent probes and then visualized with either a confocal microscope (Model LSM-510 Meta, Carl Zeiss) or a fluorescence microscope (Axio Scope inverted fluorescence microscope, Carl Zeiss). The confocal microscope images were analyzed using LSM-510 software, and the images from the fluorescence microscope were analyzed using AxioVision imaging software. Three biological replicates were used for all microscopic studies.

### Optical microscopy (OM)

For OM studies, unstained mycelia from *R. solani* that were treated with 20 μg/ml of mASAL or were left untreated (exposed to PBS only) were visualized using an Axio Scope inverted fluorescence microscope (Carl Zeiss) under bright field.

### Scanning electron microscopy (SEM)

For SEM analysis, both treated and control *R. solani* samples were spread with a sterile tip on the surface of a Peltier-cooled coolstage in a low-vacuum scanning electron microscope (Zeiss EVO-18). Fungal hyphae were investigated under low vacuum conditions (typically 0.65–0.80 mbar at 20.0 kV). The scans were recorded at 5000 × magnification.

### SYTOX Green uptake assay

The procedure and quantification of the SYTOX Green uptake assay were performed as described previously [22]. Briefly, *R. solani* cells from 3-day-old cultures were treated with either mASAL or phosphate buffered saline (PBS) or 5 μm melittin (Sigma) as positive control [23] for 24 h and then incubated with 0.8 μM SYTOX Green (Molecular Probes; Invitrogen) for 15 min in the dark. The mycelia were then washed three times with PBS, mounted in 20 % glycerol and visualized under a laser scanning confocal microscope with excitation and emission wavelengths of 488 nm and 538 nm, respectively. For the quantification of SYTOX Green uptake, approximately 200 μl of similarly treated *R. solani* hyphal suspensions was placed in a 96-well microtiter plate and incubated with 0.8 μM SYTOX Green for 15 min. SYTOX Green uptake was quantified by measuring the fluorescence emission with a microplate reader (Thermo Scientific, Varioskan Flash). The experiment was performed in triplicate and the average data are presented.

### Glucose-induced acidification assay

To determine the membrane disorganization of *R. solani*, glucose-induced acidification of the external media was measured following previously described protocol but with slight modifications [24]. Three-day-old *R. solani* mycelia were washed twice with distilled water. Approximately 1.0 g of the washed mycelia (wet weight) was resuspended in 30 ml of sterile water and incubated with mASAL (20 μg/ml) or PBS (control) for 10 min at room temperature (RT). The mycelia were filtered and incubated in 20 ml of 2 % (w/v) glucose solution with continuous stirring to induce medium acidification. The change in the external pH was measured using a digital pH meter (Hanna Instruments HI 110 Flexible Calibration pH Meter, USA) at time intervals of 0, 10, 20, 30, 40, 50, and 60 min. The average of the data from three independent sets of experiments is presented.

### Determination of K^+^ leakage

To determine the effect of mASAL on the permeability of the *R. solani* plasma membrane, a potassium release assay was performed [25]. Three-day-old *R. solani* mycelia were harvested and washed in sterile distilled water. The mycelia were then resuspended in 2 % (w/v) glucose and 16 mM glutamine. mASAL was added at concentrations of 10, 15 or 20 μg/ml and the mycelia were incubated at 22 °C for 80 min. As a negative control, the fungal hyphae were treated with water. The assay was stopped by centrifugation at 13,000 × g for 10 min, and the supernatants were collected in sterile microtubes for spectrometric analysis. The K^+^ concentration in the supernatant was measured using flame atomic absorption spectrophotometry at 766.5 nm (Systronics: Flame Photometer-130). The experiments were carried out in triplicate.

### Measurement of mitochondrial membrane potential (MMP)

The effect of mASAL on the MMP of *R. solani* was detected using the fluorescent dye Rhodamine (Rh)-123 as described previously [26]. Three-day-old *R. solani* mycelia were either treated with various concentrations of mASAL (10, 15, or 20 μg/ml) for 90 min. Control mycelia on the other hand received no mASAL treatment. As a positive control for oxidative stress induced mitochondrial membrane permeabilization we have used 30 mM H_2_O_2_ treated fungal mycelia. As hydrogen peroxide mediated change in MMP in *Penicillium expansum* has previously been reported in the literature [27] we opted for H_2_O_2_ as a known inducer of MMP in fungal cells. Rh-123 was added to a final concentration of 100 ng/ml and then the samples were incubated in the dark at RT for 30 min. After incubation, the mycelia were harvested via centrifugation at 5000 × g for 5 min and washed twice with PBS. Fluorescence was observed with a laser scanning confocal microscope with excitation at 488 nm and emission at 525 nm.

### Determination of endogenous reactive oxygen species (ROS) generation

ROS generation in mASAL treated hyphae of *R. solani* was detected using dichlorodihydrofluoresceindiacetate (H_2_DCFDA, Molecular Probes) as described by Ezaki et al. [28]. Fungal hyphae were treated with either 20 μg/ml mASAL, PBS (control) or 30 mM H_2_O_2_ (positive control) [27] followed by incubation with 100 μl of 10 μM H_2_DCFDA for 90 min. The stained hyphae were visualized under a fluorescence microscope with excitation and emission wavelengths of 488 nm and 530 nm, respectively. The images were captured with a laser scanning confocal microscope with appropriate filters according to the manufacturer’s protocol.

### DAPI staining of *R. solani* hyphal nuclei

To detect the nuclear morphology of both untreated and mASAL treated (20 μg/ml of mASAL for 24, 48 or 72 h) fungal mycelia were incubated in PBS supplemented with 1 μg/ml DAPI for 30 min at RT. The stained hyphae were then visualized with a fluorescence microscope with an excitation of 365 nm and emission of 420-540 nm.

### DNA fragmentation assay

The effect of mASAL on the integrity of nuclear DNA of *R. solani* hyphae was assayed using a DNA fragmentation assay. Genomic DNA from *R. solani* hyphae treated with 20 μg/ml mASAL for 24, 48 or 72 h and from control (i.e., treated only with PBS) hyphae was extracted by crushing the cells in presence of liquid nitrogen and incubating the ground material in 500 μl of lysis buffer (10 mM Tris pH 8.0, 100 mM NaCl, 1 mM EDTA, 1 % SDS, 2 % Triton X-100) and 500 μl of 1:1 phenol chloroform. The resulting suspension was centrifuged, and the DNA in the aqueous layer was precipitated using 100 % ethanol. Approximately 10 μg of the resulting genomic DNA was subjected to electrophoresis on a 1 % agarose gel for approximately 1.0 h at 100 V. The gel was stained with 1 mg/ml ethidium bromide and visualized by UV light on a Gel Doc system from Bio-Rad.

### Annexin-V and PI staining

Exposed phosphatidylserine in mASAL treated *R. solani* hyphae was detected using FITC-conjugated annexin V (Annexin-V FITC Apoptosis Kit, Sigma) as described by Madeo et al. [29] with some modifications. Both control (treated only with PBS) and mASAL treated (20 μg/ml for 48 h) fungal mycelia were harvested and washed with sorbitol buffer (1.2 M sorbitol, 0.5 mM MgCl_2_, and 35 mM K_2_HPO_4_, pH 6.8). The cell walls were digested with 2 % Macerozyme R-10 (Sigma) and 15 U/ml lyticase (Sigma) in sorbitol buffer for approximately 3 h at 28 °C. The cells were harvested and washed with binding buffer (10 mM HEPES/NaOH, pH 7.4, 140 mM NaCl, and 2.5 mM CaCl_2_) containing 1.2 M Sorbitol (binding-sorbitol buffer). To 96 μl hyphal suspensions in binding-sorbitol buffer, annexin V-FITC and PI are added to a final concentration of 1.2 μg/ml and 5 μg/ml respectively. The resulting suspension was then incubated at room temperature for 20–30 min. Following this the cells were immediately visualized using a confocal laser scanning microscope. A filter for FITC (excitation at 450–500 nm and emission at 515–565 nm) and PI (excitation at 550/25 nm and emission at 605/70 nm) was used. The experiments were performed in triplicate.

### Molecular target identification

#### Isolation of fungal protein

*R. solani* was grown in potato dextrose broth (PDB) at 28 °C in darkness for three days. The mycelia were collected, washed, frozen in liquid nitrogen and stored at −80 °C until further processing. The fungal protein was extracted according to Banerjee et al. [20] with some modifications [30]. 1 g lyophilized mycelium was homogenized in liquid nitrogen with a mortar and pestle and the powder was suspended in 5 ml of lysis buffer [0.05 M Tris-HCl pH 8.0, 2 % SDS, 50 mM DTT, 5 mM EDTA, 0.001 % phenylmethylsulfonylfluoride (PMSF) and 100 μl/10 ml Protease inhibitor cocktail (Sigma, St Louis, Mo)]. The mixture was vortexed thoroughly for 1 h at 4 °C and centrifuged at 20,000 × g for 20 min and the supernatant was collected. Following centrifugation the supernatant was precipitated overnight with freshly prepared 2 ml of 10 % TCA, 0.01 % DTT in pre-chilled acetone. Protein pellet was obtained by centrifugation at 20,000 × g for 30 min. The pellet was washed twice with chilled washing acetone with 0.01 % DTT and air dried. Final pellet was resuspendend in 100 μl of rehydration (IEF) buffer containing 7.0 M urea, 2.0 M thiourea, 20 mM dithiothreitol (DTT), 0.5 % bioampholytes, and 2 % 3–[(3-cholamidopropyl)-dimethylammonio]-1propanesulfonate and stored at −80 °C. Protein content was estimated using Bradford assay.

### Separation of fungal proteins by two-dimensional gel electrophoresis (2-DE)

Two-dimensional gel electrophoresis (2-DE) was performed to obtain the gel profile of the fungal (*R. solani*) mycelial proteome. 120 μg of fungal protein were solubilized in rehydration buffer (IEF). A total of 125 μL of IEF buffer was applied to 7 cm (pH4 − 7) IPG strips (BioRad, CA, USA) and left overnight for passive rehydration after overlaying with mineral oil (BioRad, CA, USA). After incubation, the strips were transferred to the focusing tray. Paper wicks were placed at both the ends of the channels of focusing tray covering the wire electrodes, followed by the addition of 8 μl of nanopure water on each wick to wet them. The strips were covered with mineral oil and the separation of proteins in the first dimension was performed in an IEF cell (BioRad, CA, USA) by using the standard program: The strips were focused at 250 V for 20 min, 4000 V for 2 h with linear voltage amplification and finally to 10,000 V hour with rapid amplification. After focusing, the strips were reduced and alkylated for 15 min each, using equilibration buffer-I (6 M Urea, 75 mM Tris-Cl pH 8.8, 30 % glycerol, 2 % SDS and 1 % w/v DTT) and equilibration buffer-II (same as equilibration buffer-I with 2.5 % w/v iodoacetamide instead of DTT) respectively. After equilibration the strips were held in position with overlay agarose (BioRad, CA, USA). Finally, strips were run in hand-cast 12 % SDS-PAGE (7 cm × 10 cm × 1 mm) with the Bio-Rad Mini-PROTEAN 3 electrophoresis system at a constant volt (200 V,500 mA,99 W) for 1 h in tris-glycine SDS running buffer (250 mM glycine, 25 mM Tris and 0.1 % SDS) until the dye front reached near the bottom edge of the gel. Gels were stained with staining solution [10 % Coomassie Brilliant Blue -G250 (w/v); 50 % methanol (v/v); 7 % glacial acetic acid (v/v)] at room temperature, for 1 h and subsequently destained with destaining solution (2.5 % methanol, 10 % acetic acid) with gentle agitation in a rocker platform.

### Ligand blot assay

The mycelial proteome of *R. solani* was resolved in a 2-DE gel and electrophoretically transferred onto a Hybond-C membrane (GE Healthcare) with a blotting buffer (39 mM glycine, 48 mM Tris base, 20 % methanol, and 0.037 % SDS) using a semidry blotting apparatus (TE77; Amersham Pharmacia Biotech). The electrotransfer was run for 60 min at a current of 56 mA, 25 V. The membrane was temporarily stained with Ponceau S (Sigma-Aldrich, USA) to ensure the protein transfer from gel to Hybond-C membrane. The membrane was incubated for 15 min in Ponceau S staining solution with gentle agitation. Finally the membrane was rinsed in distilled water for two washes of 5 min each until the background is clean. Then the membrane was blocked overnight in 10 ml blocking buffer [5 % nonfat milk (Merck, Germany) in 1 × TBST]. Next day, the membrane was washed with three changes of TBST for 2 min each time and further incubated with mASAL (20 μg) for 2 h at 37 °C. Finally, the blot was incubated using a primary anti-mASAL polyclonal antibody (1:8000) and an anti-rabbit IgG HRP-conjugated secondary antibody (1:20,000, Sigma-Aldrich, USA). Membranes incubated without mASAL served as negative controls (data not shown).

### In-gel digestion of putative interacting proteins

The previously alkylated and reduced 2-DE Coomassie-stained protein spots corresponding to the ligand blot signals were excised manually and subjected to in-gel tryptic digestion for mass spectrometry analysis following the protocol of Shevchenko et al. [31] with minor modifications. The gel pieces were destained and then freshly prepared porcine trypsin (Promega, USA) solution (10 mM NH_4_HCO_3_/5 % CH_3_CN with 5 ng/μl of trypsin) was added to cover the gel pieces. In gel digestion was carried for 16 h at 37 °C in a water bath. The peptides were extracted with 25 % acetonitrile and 1 % trifluroacetic acid. Finally, the tryptic peptides were extracted, vacuum dried and frozen prior to MS analysis.

### Mass spectrometric identification of putative interacting proteins

The extracted peptides were analyzed by capillary liquid chromatography tandem mass spectrometry with an EASY-nLC 1000 using the two column set up (Thermo Scientific). The peptides were loaded in buffer A onto a peptide trap (Acclaim PepMap 100, 75um × 2 cm, C18, 3um, 100 Å) at a constant pressure of 500 bar. Then they were separated, at a flow rate of 200 nl/min with a linear gradient of 2–30 % buffer B in buffer A in 20 min followed by an linear increase from 30 to 50 % in 5 min (Buffer A: 0.1 % formic acid, buffer B: 0.1 % formic acid in acetonitrile) on a 75um × 15 cm ES800 C18, 3um, 100 Å column mounted on a DPV ion source (New Objective) connected to a Orbitrap Velos (Thermo Scientific). The data were acquired using 60,000 resolution for the peptide measurements in the Orbitrap and a top 20 method with CID fragmentation and fragment measurement in the LTQ, or a HCD top 6 with measurement in the Orbitrap with 7500 resolution for the fragment measurement was used, according the recommendation of the manufacturer. Mascot 2.3 (Matrix Science, London, UK) searching UniProt data base version 2013_11 (45288084 entries) was used to identify the peptides. The enzyme specificity was set to trypsin allowing for up to three incomplete cleavage sites. Carbamidomethylation of cysteine (+57.0245) was set as a fixed modification, oxidation of methionine (+15.9949 Da) and acetylation of protein N-termini (+42.0106 Da) was set as variable modifications. Parent ion mass tolerance was set to 5 ppm and fragment ion mass tolerance to 0.6 Da. Decoy search was performed to avoid false identification of peptide by matching it to a random sequence from a decoy database and the desired protein false discovery rate (FDR) cut off was set at 0.01. The results were validated with the program Scaffold Version 4.0 (Proteome Software, Portland, USA). Peptide identifications were accepted if they could be established at greater than 95.0 % probability as specified by the Peptide Prophet algorithm [32] with Scaffold delta-mass correction were considered. Protein identifications were accepted if they could be established at greater than 95.0 % probability and contained at least 5 identified peptides. Protein probabilities were assigned by the Protein Prophet algorithm [33].

### Co-immunoprecipitation of candidate mASAL interactors

For co-immunoprecipitation of potential mASAL interacting proteins the total cell lysate from *R. solani* cells were prepared as described before. One ml of cell lysate was incubated with 100 μg of purified recombinant mASAL at 4 °C overnight. Equilibrated Ni-NTA-agarose beads (Qiagen, Germany) were added to each lysate - protein mixture, further the reactions were allowed to rock slowly at 4 °C for 1 h. The beads were pelleted at 3000 × g for 10 min. The supernatant was discarded and the beads were washed twice with 500 μl of lysis buffer. Following this the beads were finally resuspended in 40 μl of 1X SDS-PAGE loading buffer and boiled for 10 min. After boiling the samples were centrifuged and the eluted proteins were separated by SDS-PAGE and immunoblotted onto a nitrocellulose membrane (Hybond-C, GE Healthcare). After blocking, the membranes were probed with primary antibodies against either ATPase or HSP70 or Actin (Pierce, USA). Following this each of the blots were incubated with anti-mouse IgG conjugated to horse radish peroxidase (HRP) (Sigma-Aldrich, USA) at 1:20,000 dilutions. Bands were detected by enhanced chemiluminescence (ECL) reagents (GE Healthcare, Germany).

### Identification of functional partners of mASAL interactors using STRING database

The functional partners of each of the identified mASAL-interacting proteins were predicted using a precomputed protein-protein interaction database (STRING version 9.0, http://string-db.org) [34]. Because the database lacks information on the *R. solani* proteome, homologs of the candidate interacting proteins from either *Saccharomyces cerevisiae* or *Homo sapiens* were analyzed. In each individual case, hits showing a confidence score of 0.5–0.9 were considered. The available information in the database about the predicted functional partners of the interacting proteins was used to determine the cellular pathways that might be affected by mASAL treatment of *R. solani.*

### Statistical analysis

For all assays, three independent experiments were carried out. Two tailed P values of less than 0.05 were considered to be statistically significant.

## Results

### The effect of mASAL on the hyphal morphology of *R. solani*

**Fig. 1:**
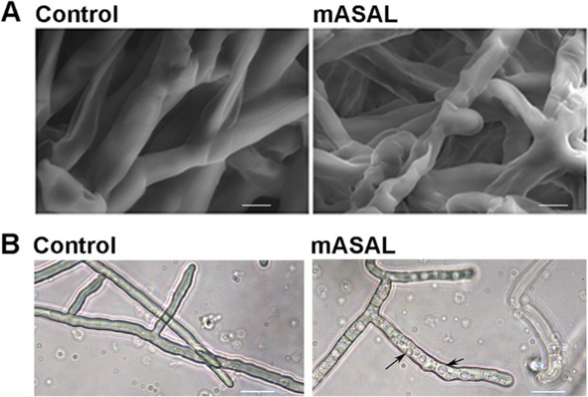
Microscopic analysis of *R. solani*. **a** Scanning electron micrographs of *R. solani* treated with either PBS buffer (*left panel*) or 20 μg/ml mASAL (*right panel*). Scale bars represent 6 μm. **b** optical microscopy of *R. solani* after incubation with either PBS as control (left panel) or 20 μg/ml mASAL (right panel). Arrow indicates intracellular vacuole. Scale bars represent 25 μm

Scanning electron microscopy of *R. solani* hyphae treated with mASAL revealed significant changes in the structure of the cell. In contrast to untreated hyphae, whose cell walls appeared smooth in texture, the cell walls of mASAL-treated hyphae had a wrinkled appearance (Fig. 1a). In addition, light microscopy showed that mASAL treatment was associated with extensive intracellular vacuolization (Fig. 1b).

### mASAL-treated *R. solani* is permeable to SYTOX Green

**Fig. 2:**
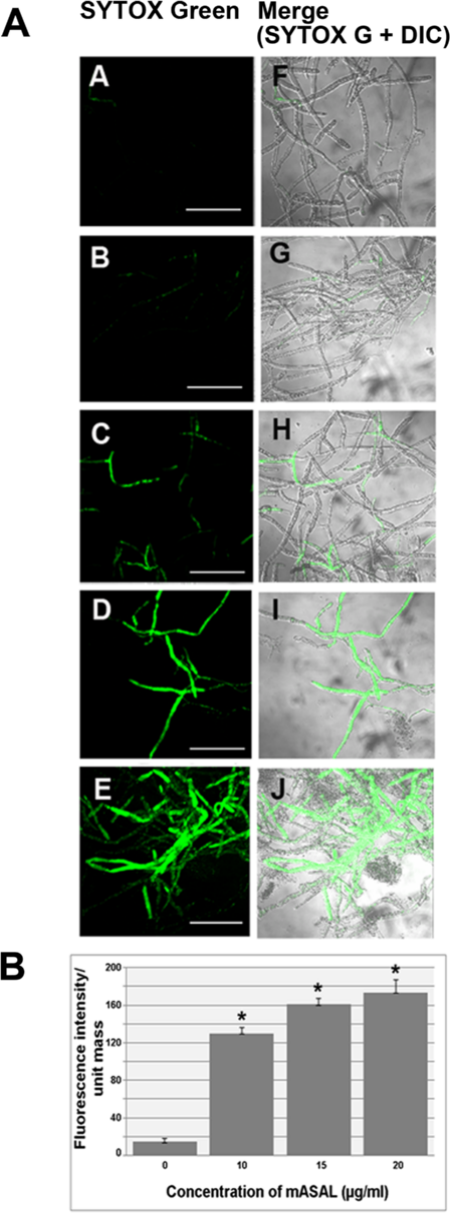
SYTOX Green uptake by *R. solani* hyphae. **a** Confocal laser scanning microscopic images of *R. solani* hyphae treated with mASAL. *R. solani* mycelia were incubated in half-strength PDB at 28 °C without mASAL (panel ***a*** and ***f***) or in the presence of mASAL at final concentrations of 10 μg/ml (panels ***b*** and ***g***), 15 μg/ml (panels ***c*** and ***h***), or 20 μg/ml (panels ***d*** and ***i***). As a positive control *R. solani* hyphae treated with 5 μM melittin, another antimicrobial peptide is shown in panels ***e*** and ***j***. Panels ***a***, ***b***, ***c***, ***d*** and ***e*** represent fluorescent images while panels ***f***, ***g***, ***h***, ***i*** and ***j*** represent merged images with DIC (differential interference contrast). Microscopy of SYTOX Green uptake on mASAL treatment was done in three independent sets and the representative image is presented. Scale bars represent 50 μm. **b** Quantification of SYTOX Green uptake by mASAL treated cells. Fungal mycelia were treated with increasing concentrations of mASAL (0, 10, 15, 20 μg/ml) for 24 h prior to incubation with SYTOX Green. Permeabilization was quantified by SYTOX Green uptake. Each value represents the average of three independent experiments with standard deviations as error bars (*, *P* < 0.05)

To assess the effect of mASAL on the plasma membrane permeability of *R. solani*, a SYTOX Green-based uptake assay was designed. In the present study, confocal microscopic analysis of *R. solani* hyphae incubated in SYTOX Green without mASAL pretreatment showed no appreciable fluorescence. However, pretreatment with mASAL led to a significant increase in the intracellular fluorescence signal, indicating the uptake of the dye. Moreover, the intensity of the signal increased with increasing concentrations of mASAL (Fig. 2a). The effect was found to be comparable to treatment of *R. solani* hyphae with melittin, another antimicrobial peptide that has been previously shown to induce membrane permeabilization in *Penicillium digitatum* [23]. Melittin was therefore used as a positive control in this experiment to study induction of plasma membrane permeabilization by mASAL. Quantification of SYTOX Green uptake further confirmed that the increase of the permeabilization of the *R. solani* plasma membrane was dependent on increasing the concentration of mASAL used to treat the fungus (Fig. 2b).

### mASAL induces acidification of the external media

**Fig. 3:**
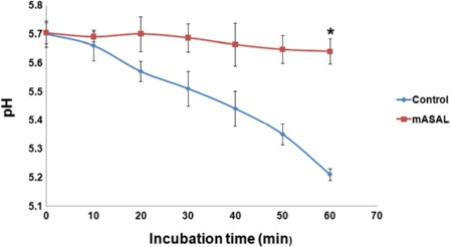
Glucose-induced acidification of medium by *R. solani* upon mASAL treatment. The mycelia of *R. solani* were washed and resuspended in sterile water and challenged with mASAL (20 μg/ml) (squares) or water (diamond) followed by resuspension in 2 % (w/v) glucose solution. The change of the external pH was measured using a digital pH meter at time points of 0, 10, 20, 30, 40, 50, and 60 min. Each value represents the average of three independent experiments with standard deviations as error bars (*, *P* < 0.05)

In the presence of glucose, many fungi can acidify the external medium by pumping out protons through the plasma membrane H^+^ ATPase [24, 35]. Acidification of fungal growth medium is therefore an indication that the organism possesses an intact, healthy plasma membrane. Media acidification by *R. solani* was significantly reduced after treatment with mASAL. In contrast to untreated cells, which could lower the external pH from 5.75 to approximately 5.2 after 1 h incubation in 2 % glucose, the treated cells reduced the pH by approximately 0.05 units, with a final pH of approximately 5.7 (Fig. 3).

### mASAL treatment leads to K^+^ leakage across the *R. solani* plasma membrane

**Fig. 4:**
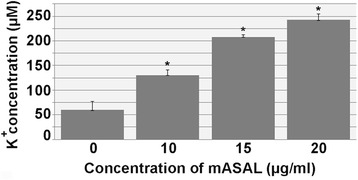
K^+^ release in the supernatant of *R. solani* cultures. Cells of *R. solani* were challenged with different concentration of mASAL (0, 10, 15 and 20 μg/ml) as indicated. Potassium release in *R. solani* was measured using flame atomic absorption spectrometry (FAAS). Each data point represents the average of three individual measurements with standard deviations as error bars (*, *P* < 0.05)

The effect of mASAL on the cell function and plasma membrane integrity of *R. solani* was further studied by measuring potassium efflux. Potassium release was studied after an incubation period of 80 min. As shown in Fig. 4, mASAL-treated cells released more potassium ions than did untreated cells. *R. solani* cells treated with 10 μg/ml mASAL released twice as much K^+^ as untreated cells. The release of K^+^ also increased with increasing concentrations of mASAL.

### The effect of mASAL on mitochondrial membrane potential (MMP)

**Fig. 5:**
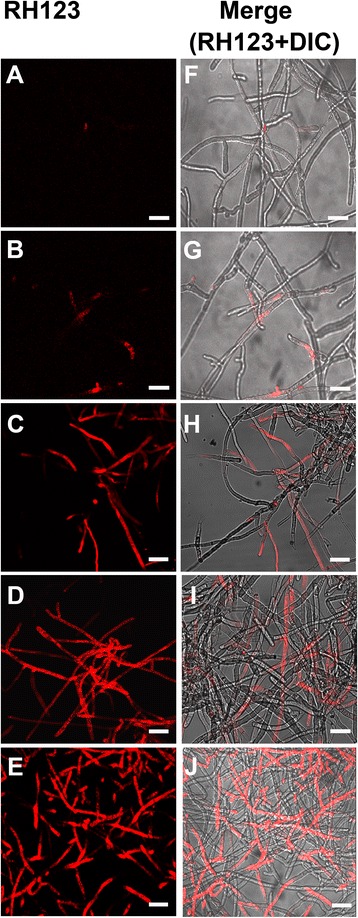
Confocal laser scanning micrographs of *R. solani* cells stained with Rh123. *R. solani* hyphae either untreated (panels **a** and **f**) or treated with 10 μg/ml (panels **b** and **g**), 15 μg/ml (panels **c** and **h**), 20 μg/ml (panels **d** and **i**) mASAL or 30 mM H_2_O_2_ (panels **e** and **j**) were stained with Rh123 to assess mitochondrial membrane potential. Left column represents fluorescent images and right column represents merged images with DIC (differential interference contrast). Scale bars represent 50 μm

MMP is a very sensitive indicator of the energetic state of the mitochondria. It can also be used to monitor the activity of mitochondrial proton pumps and electrogenic transport systems, as well as the transition to mitochondrial permeability. Rh123 is a potential-dependent distribution probe that preferentially enters the mitochondria under highly negative MMP. The results presented in Fig. 5 show a decrease in the MMP of *R. solani* with increasing mASAL concentration, as evidenced by an increase in the fluorescence of the dye in treated cells compared to untreated cells. In order to get an idea of the degree of loss of MMP in response to mASAL treatment of *R. solani* hyphae we used a previously established oxidizing agent, hydrogen peroxide (H_2_O_2_) [27]. Confocal microscopic images of *R. solani* hyphae treated with 30 mM H_2_O_2_ showed comparable fluorescence intensities to that of the mASAL treated cells indicating that mASAL possibly exhibits similar detrimental effects on MMP as that of H_2_O_2_.

### mASAL induces endogenous ROS production

**Fig. 6:**
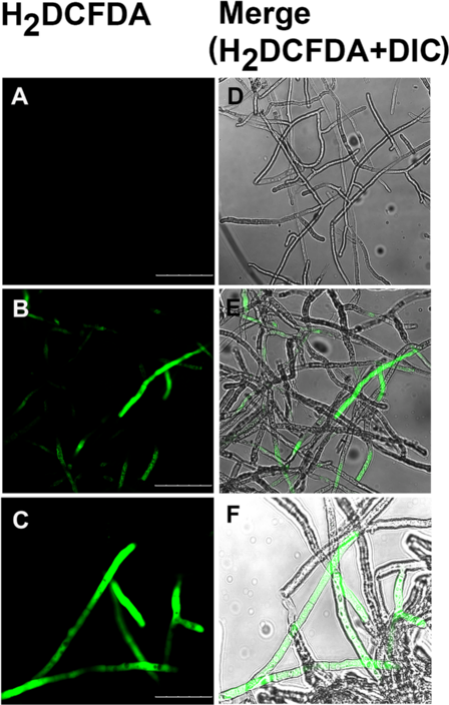
Endogenous ROS production on mASAL treatment in *R. solani* hyphae detected by H_2_DCFDA. Hyphae were either treated with 20 μg/ml mASAL for 90 min (panels **b** and **e**), untreated that serve as control (panels **a** and **d**) or 30 mM H_2_O_2_ (panels **c** and **f**). Panels **a**, **b** and **c** represent fluorescent images and panels (**d**, **e** and **f**) represent DIC (differential interference contrast) images. Scale bars represent 25 μm

The fluorescent dye H_2_DCFDA was used to investigate intracellular ROS production in mASAL-treated *R. solani* hyphal cells. As shown in Fig. 6, compared to untreated cells (panel A) significant fluorescence was detected in mASAL-treated hyphal cells (panel B). ROS-specific signals were observed throughout the hyphae along the plasma membrane and within the cytoplasm surrounding the vacuole. Similar distribution of fluorescent signals could also be obtained in case of *R. solani* hyphae treated with 30 mM H_2_O_2_ (positive control) [27].

### mASAL induces PCD in *R. solani*

**Fig. 7:**
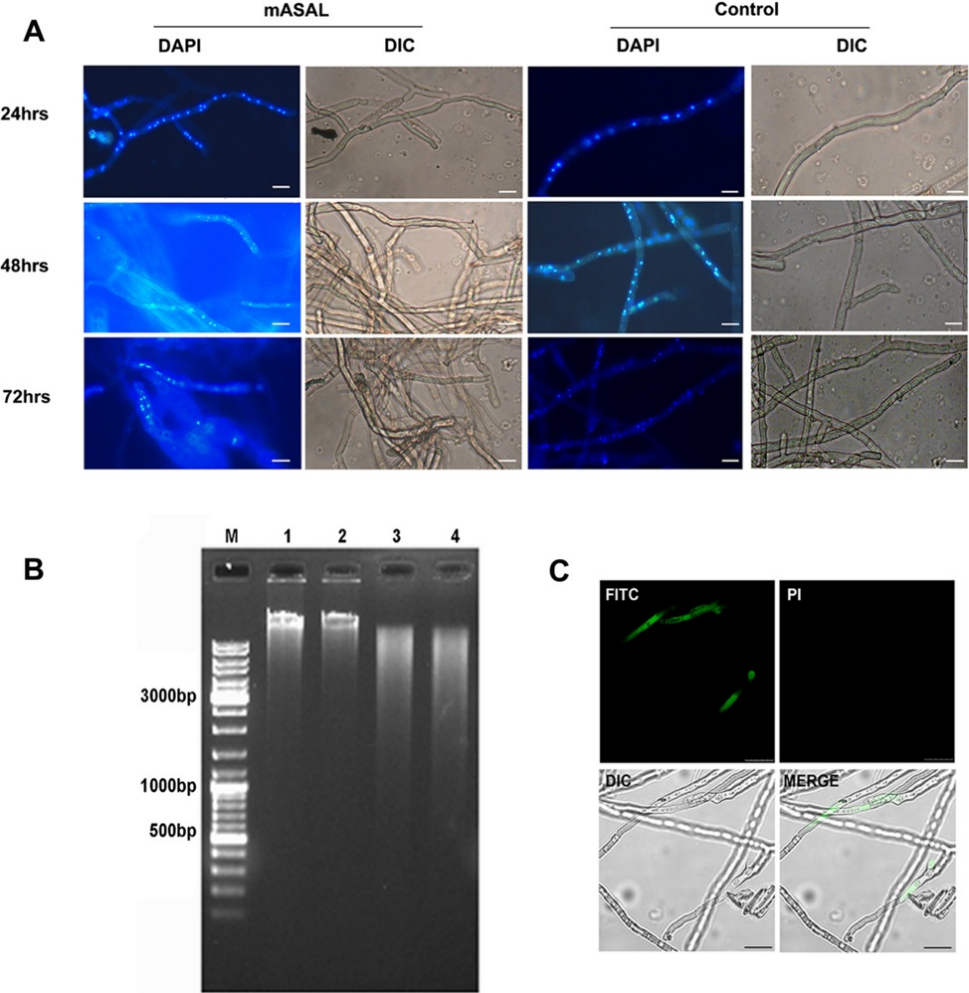
mASAL induced PCD in *R solani*. **a** Nuclear morphology of *R. solani* by DAPI staining. First column: *R. solani* hyphae treated with 20 μg/ml mASAL for different time intervals, 24 h (upper panel), 48 h (middle panel) and 72 h (lower panel). First and third column represent the DAPI stained *R. solani* hyphae treated with mASAL and PBS (control) respectively. Second and fourth column represent the DIC (differential interference contrast) images of mASAL treated and control sample respectively. Scale bars represent 20 μm. **b** DNA fragmentation induced by mASAL. Genomic DNA from *R. solani* cells treated with either 20 μg/ml mASAL for three different time points or PBS for control were run on 1 % agarose gel. Lane 1: DNA of *R. solani* treated with PBS buffer for 72 h as the control. Lane 2–4: DNA of *R. solani* treated with mASAL for 24, 48 and 72 h respectively. Lane M represents DNA molecular weight marker. **c** Annexin-V-FITC assay. The mycelia of *R.solani* were treated with 20 μg/ml mASAL for 48 h and stained with annexin-V-FITC and Propidium iodide (PI). Upper Left panel: Annexin V-FITC, upper right panel: PI, Lower left panel: DIC (differential interference contrast), Lower right panel: Merge images of FITC/PI and DIC. Bars represent 25 μm

Treatment of *R. solani* hyphae with mASAL was associated with the induction of PCD. DAPI staining of the nuclei of the treated hyphae revealed prominent DNA condensation and fragmentation (Fig. 7a), a typical hallmark of apoptotic cell death. After 24 h, the nuclei of the treated and untreated cells could be observed as intense, discrete signals, whereas after 48 h, the nuclei of the treated cells showed faint signals (Fig. 7a) and were much smaller. The effect was even more prominent after 72 h; where in most of the hyphae did not show discrete nuclear signals. A quantitative analysis of the number of intact nuclei present in hyphal tip cells of either untreated or mASAL treated hyphae also supported the above observation. For instance the number of intact nuclei in hyphal cells treated with mASAL for 72 h is approximately 1/10^th^ of that of the untreated cells (Additional file 1). The effect of mASAL treatment on the nuclear DNA of *R. solani* was further confirmed by carrying out an in-gel DNA fragmentation assay with genomic DNA (gDNA) extracted from both the treated and untreated hyphae. Compared with untreated hyphae, the gDNA from the treated hyphae appeared to be degraded, yielding a smear in the agarose gel (Fig. 7b). Moreover, the treated hyphae also showed positive staining with annexinV-FITC (Fig. 7c) thereby further supporting mASAL mediated probable apoptotic death of fungal cells. Nevertheless, light microscopic analysis of mASAL treated hyphae exhibited much increased intracellular vacuolization than the untreated samples. In contrast to untreated hyphae, which showed very few intracellular vacuoles, the treated hyphae underwent extensive vacuolization (Fig. 1b). Whether this increased vacuolization is an indication towards other forms of programmed cell death operative in the pathogen in response to mASAL treatment is yet to be studied in details.

### Identification of mASAL-interacting proteins from *R. solani*

**Fig. 8:**
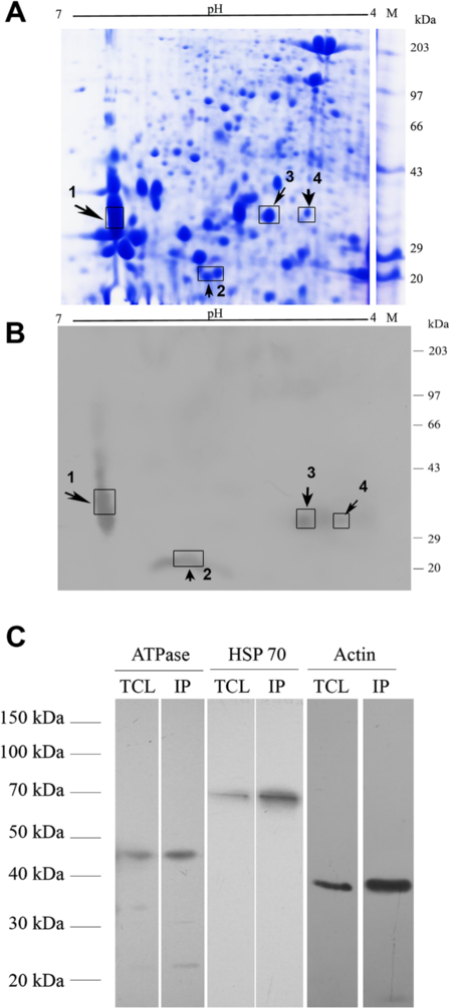
Identification of potential interactors of mASAL. **a** Representative 2-DE profile of the total protein extracted from *R. solani* in the pH range of 4–7 stained with Coomassie Brilliant Blue R-250 (**b**) Ligand blot of the same gel challenged with mASAL with subsequent incubation with mASAL specific antibody and respective secondary antibody. Arrows and boxes indicate spots with similar positioning in both A and B that are selected for analysis using LC MS/MS. M represents molecular weight marker. **c** Western blots showing co-immunoprecipitation of *R. solani* ATPase (lane 2), HSP 70 (lane 4) and Actin (lane 6) with recombinant mASAL from the total cell lysates of the pathogen. Lanes 1, 3 and 5 show the specificity of the antibodies used for ATPase, HSP 70 and Actin recognition respectively in *R. solani* total cell lysates. In each of the three cases mentioned above, the antibodies recognized single targets observed as single bands at expected size

A ligand blot of 2-D gel was performed by incubating mASAL with a blot containing total fungal protein and detecting the bound mASAL with an anti-mASAL antibody (Fig. 8). The tryptic peptide fragments were analyzed by tandem mass spectrometry (LC-MS/MS), and each MS/MS spectrum was searched against the UNIPROT _131112 database. Total peptides fragments found by LC MS/MS analysis of the ligand positive spots are provided in the Additional file 2. The confidence of identification of protein was based on following criteria: (1) identification with the target organism, *R.solani* (2) number of unique matched peptides specific to the particular protein and (3) percentage of sequence coverage (4) total spectral count. The interacting proteins that were identified were Actin, HSP70, ATPase and 14-3-3 from *Thanatephorus cucumeris* (Table 1). Details of the identified proteins are in the Additional file 3. In order to further confirm these proteins as the potential interactors of mASAL a co-immunoprecipitation assay was performed following incubation of recombinant mASAL with the total cell lysates from *R. solani.* In this experiment Ni-NTA-agarose was used for precipitating mASAL. A western blot analysis using these immunoprecipitates using antibodies against each of the identified interactors revealed single bands both in the total cell lysates as well as in the immunoprecipitates (Fig. 8c).

**Table 1:** Interacting partners of mASAL identified through LC MS/MS analysis

Spot no/zone	UniPort accession no	Interacting partners	Organism^a^	Obs kDa/Exp kDa	Amino acid match	Sequence coverage
1	L8X715_THACA	HSP70	*Thanatephorus cucumeris*	40 kDa/180 kDa	110/1685	7 %
2	L8X4T0_THACA	14-3-3	*Thanatephorus cucumeris*	20 kDa/68.3 kDa	104/615	17 %
3	L8WKN1_THACA	Actin	*Thanatephorus cucumeris*	35 kDa/70.9 kDa	78/633	12 %
4	L8WKN1_THACA	ATP synthase subunit beta	*Thanatephorus cucumeris*	35 kDa/64.2 kDa	165/597	28 %

^a^The sexual stage of *Rhizoctonia solani* is known as *Thanatephorus cucumeris*

## Discussion

Because the invasion of fungal diseases and the development of resistance to the target pathogens are becoming more prevalent in agriculture [36], the search for novel antifungal agents is of considerable interest. However, the sustainable management of fungal diseases requires complete knowledge of the mechanisms of action of the novel antifungal agents, including the identification of their molecular targets. To fully harness the potential of mASAL for bioengineering crops for developing robust resistance to *R. solani* infection, it is necessary to understand the mode of action of this unique antifungal protein. Therefore, we attempted to gain insight into the mechanism of action of mASAL on the growth and development of *R. solani*.

### Alterations in hyphal morphology

Ultrastructural studies using scanning electron microscopy showed prominent distortion of the mASAL-treated mycelia, which appeared wrinkled and collapsed compared to the untreated mycelia. This observation indicates a possible interaction between mASAL and components of the *R. solani* cell wall, potentially leading to structural disruption of the cell. Alternatively, the data might also give us an indication of the activation of certain intracellular signaling pathways the end result of which involves structural disruption of the fungal cell.

### The loss of plasma membrane integrity and function

In addition to affecting the cell wall, mASAL was also found to affect the permeability of underlying plasma membrane. The plasma membrane plays a pivotal role in the maintenance of homeostasis between the cellular interior and the exterior milieu by regulating the transport of materials. Therefore, any change in the selective permeability of the plasma membrane could have fatal consequences for the entire cell. Several studies have suggested that the ability to alter membrane permeability is one of the major functional attributes of different antifungal agents [37, 38]. SYTOX Green uptake assay, which is widely used to monitor the membrane-permeabilizing activities of different antifungal peptides [22, 23] has been used in this study to assess the ability of mASAL in the permeabilization of *R. solani* plasma membrane. Confocal microscopy clearly showed that mASAL-treated fungal hyphae were permeable to SYTOX Green whereas untreated cells remained impermeable. Moreover, the quantification of SYTOX Green uptake revealed that the permeability of the fungal plasma membrane increased with increasing concentrations of mASAL. In a recent study, a plant-derived lectin was shown to have a similar effect on the membrane permeability of *Candida tropicalis*, *Pichia membranifaciens*, and *Candida albicans* [39]. The probable disruption of the *R. solani* plasma membrane by treatment with mASAL was supported by the inhibition of glucose-induced media acidification. In healthy cells, the presence of an energy source like glucose induces plasma membrane ATPases to carry out proton efflux, leading to media acidification. Any direct or indirect damage to the plasma membrane ATPases can result in the inhibition of this phenomenon and a subsequent decrease in the extracellular concentration of H^+^ ions. Therefore, our data suggest that mASAL has a detrimental effect on these ATPases that is most likely caused by disruption of the plasma membrane structure. A similar ability to inhibit glucose-induced medium acidification has been reported previously by various antifungal compounds [24, 40]. Alternatively, mASAL could also affect the function of mitochondrial ATPases, causing the depletion of large amounts of cellular ATP, which is required to fuel the plasma membrane ATPases. As a result, the proton pumping function of the plasma membrane ATPases could be affected, leading to inhibition of the acidification of the extracellular media [24]. The effect of mASAL on the permeability of the plasma membrane was further confirmed by a significant release of potassium ions from the mASAL-treated hyphae of *R. solani*. In yeast cells potassium release was triggered when exponentially growing yeast cells were challenged with 100 μg/ml of the peptidomimetic LTX109 [40]. In addition, an antifungal protein PAF was found to release the elevated amount of potassium from *A. nidulus* at concentration of 10 μg PAF/ml [25] compared to that by 10 μg/ml of mASAL, which suggests that mASAL also permeabilizes intracellular membranes.

Thus, the effect of mASAL on the integrity of the plasma membrane could be due to direct interaction with various membrane components, disruption of the lipid bilayer or indirectly through the generation of various oxidizing agents. However, it is not yet possible to clearly determine whether the effect of mASAL on the plasma membrane is a primary or secondary effect.

### Intracellular ROS generation, mitochondrial membrane permeabilization and PCD

Membrane permeabilization may also occur as a result of the generation of intracellular reactive oxygen species (ROS). Oxidative radicals are known to disintegrate the phospholipid residues of membranes via peroxidation [41]. In eukaryotic cells, the mitochondria are major generators of ROS [42]. ROS production is generally initiated by various stress-inducing factors such as irradiation and cytotoxic molecules, resulting in growth inhibition and cell death [43]. The accumulation of intracellular ROS may have a severe effect on cells, causing the random oxidation of biopolymers and the destruction of membranes and cell organelles such as mitochondria [44]. In our experiment, the fluorescent probe H_2_DCFDA was used to monitor ROS generation in mASAL-treated *R. solani*, and the results clearly confirmed that mASAL was able to induce oxidative stress through intracellular ROS accumulation in the hyphal cells. Nevertheless, ROS accumulation can also be induced by a change in MMP. In healthy cells, MMP plays an important role in the production of energy (ATP) [45]. Inhibitors of the mitochondrial electron transport chain decrease the MMP by inhibiting the proton pumping activity of the respiratory chain, resulting in a decrease in ATP and ultimately leading to cell death [46]. Therefore, the above data indicate a probable effect of mASAL on MMP, potentially causing ATP depletion and subsequent mitochondrial dysfunction. The intracellular accumulation of ROS is considered an important PCD-inducing stimulus in both lower and higher eukaryotes [47, 48]. In the present study, the evidence of the induction of PCD in mASAL-treated *R. solani* cells is reported. Many antifungal agents are reported to induce PCD via ROS generation and accumulation in filamentous fungi including *Rhizoctonia, Fusarium,* and *Aspergillus* [49–51]. Our data suggest that treating *R. solani* with mASAL may induce both an apoptotic pathway, which is evident from the nuclear fragmentation assay and annexin V-FITC assay. Extensive vacuolization of the hyphae is considered as a typical hallmark of PCD [52]. Similar examples of lectin-induced apoptotic cell death in different tumor cell lines have been reported by other groups [53, 54]. Nevertheless, a detailed investigation of the downstream components of these pathways is beyond the scope of this study. Further analysis is necessary to understand the exact signaling mechanism leading to PCD in mASAL-sensitive fungi.

### Putative interactors of mASAL

To determine the molecular basis behind the generation of ROS and the subsequent induction of PCD in *R. solani* cells following treatment with mASAL, a ligand blot analysis followed by LC-MS/MS was performed. This experiment resulted in the identification of Actin, HSP70, ATPase and 14-3-3 as candidate mASAL-interacting proteins. However, there is a difference in the observed molecular weight from its predicted molecular weight of the putative interactors. This may have occurred due to alternative splicing, proteolytic cleavage, or post-translational modifications (PTM) [55, 56]. The possibilities of artefactual proteolysis during processing or in vivo lysis of the protein also cannot be ruled out.

Though the exact mechanism of ligand binding with mASAL could not be revealed through this study, some binding features presumably be anticipated by comparing with other mannose binding dimeric lectin. ASAL is reported to recognize several receptor proteins in the midgut of different sap sucking insects [57, 58]. Glycoprotein-specific staining revealed the glycosylated nature of the ASAL-binding proteins. ASAL failed to bind with the deglycosylated midgut brush bordered membrane vesicle (BBMV) proteins [57]. In a previous study by this group [20] putative receptor of mASAL from *R .solani* was detected by one dimensional ligand blot assay. The carbohydrate-specific staining of the putative receptor protein depicted through gel analysis established the fact that individual interactors are glycoproteins. Such interactor, when deglycosylated and further analyzed through a ligand blot experiment with anti-mASAL antibody, failed to recognize and bind to mannose-specific mASAL, supporting their glycosylated characteristics. In addition, an *in silico* docking of another mannose binding insecticidal lectin *Colocasia esculenta* tuber agglutinin (CEA) with its putative interactors revealed the presence of more than one putative N-glycosylation sites located at the site of interaction or at its close proximity [59]. These observations suggested that mASAL binding with its putative interactors might have followed some glycosylation mediated binding.

**Fig. 9:**
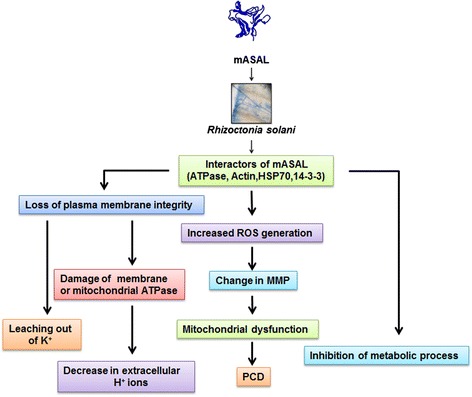
Working model showing the probable mode of action of the antifungal protein mASAL on *R. solani*. The figure depicts the detrimental effect of mASAL on various cellular components of *R. solani*. The resulting downstream changes are also schematically represented that include change in plasma membrane integrity, induction of increased intracellular ROS generation, change in mitochondrial membrane potential (MMP) leading to PCD. mASAL may also bind to putative interactors and thereby interfering with normal metabolic pathways

At this stage of the study however it is hard to predict the mechanistic details by which these identified candidate targets can contribute to the antifungal property of mASAL. Nevertheless knowledge about the usual functions of these proteins can aid in hypothesizing certain scenario. For instance Actin cytoskeleton is known to be involved in various intracellular processes like motility, vesicular trafficking and cell wall remodeling in eukaryotes. Therefore agents that could interfere with the Actin dynamics within a cell can be expected to have fatal effects. mASAL might function in a similar way by disturbing the Actin dynamics of *R. solani* through its direct interaction with Actin. HSP70 on the other hand plays major role in regulating intracellular protein quality control and induction of programmed cell death. Interference in its function therefore might lead to disruption of intracellular homeostasis leading to cell death. Similar interaction of HSP70 from insects and their bacterial endosymbiont with other lectins like *Colocasia esculenta* tuber agglutinin (CEA) and ASAL has been reported earlier by our group [58, 59]. ATPase is another key molecule controlling the energy requirements of the cell. This particular target of mASAL therefore could actually alter the ion gradient across various intracellular membranes and plasma membrane of *R. solani* following its interaction with mASAL and thereby can bring about the lethal effect. 14-3-3 is another very important signaling molecule that participates in several different intricate signaling pathways. Functional alterations of the protein therefore can be expected to have effects on many different cellular processes. The identified interactors of mASAL coincidentally are key intermediate molecules of several important metabolic processes. A search for the predicted functional partners of these proteins was therefore performed using the STRING (version 9.1) database of protein-protein interactions [34]. Due to the unavailability of *R. solani* protein data in the STRING database we selected the respective homologous proteins from either yeast or human. Since most of these protein targets identified in the study are mostly conserved across organisms this should atleast give us a glimpse of the probable events that might have happened in response to mASAL treatment on *R. solani.* The analysis however revealed a number of hits as listed and shown in Additional file 4: Figure S1 and Additional file 5: Table S2 respectively in case of each of the identified interactors. The binding affinity of mASAL to the identified key receptors might be affecting the normal metabolic pathways and thus bringing about the toxic effect of mASAL. The major pathways that are most likely to be affected therefore include cellular growth and development, cytoskeletal reorganization, regulation of programmed cell death and cell cycle, vacuolar transport of different substrates and protein homeostasis. In Fig. 9, we present a tentative working model of the antifungal activity of mASAL on *R.solani*.

mASAL seems therefore to exert its effect through interfering with different key metabolic pathways of *R. solani*. Although this mode of action of the peptide could be hypothesized for other fungi like *F. oxysporum* and *A. brassicola* that are susceptible to mASAL treatment, it can very well be interpreted from all the present data that the antagonistic mechanism of mASAL is highly specific to *R. solani*. This however needs further investigation and comparative analyses.

## Conclusions

In the present study, the antifungal activity of an indigenously designed lectin like protein, mASAL, was demonstrated. In addition, an attempt was made to decipher its mode of action by identifying candidate interacting proteins from *R. solani* proteome. However, further studies are essential to dissect how the cellular functions are altered due to blockage of the identified interactive partners. This knowledge could provide a suitable platform for the development of transgenic crops that are resistant to *R. solani* infection. Moreover, the outcomes of these studies may be instrumental in designing novel agents with stronger and more specific activity against plant pathogenic fungi.

## Additional files

Additional file 1: Figure S1. Nuclei counts in hyphae of control and mASAL treated fungal hyphae. Hyphae were harvested and stained with DAPI. The number of nuclei between the hyphal tip and the first two septum was counted manually. For the control type and mASAL treated sample, the graph shows the average number ± S.D. of nuclei counted in at three different time points (*, *P* < 0.05). (DOCX 636 kb) Additional file 2: Table S1. Excel spreadsheet showing total peptides fragments used to identify proteins. (XLSX 43 kb) Additional file 3:
**LC MS/MS analysis of identified interactors of mASAL from** ***R.solani*** **Matched peptides highlighted in yellow.** Green colour indicated probable sites of mutation and modification. (DOCX 2225 kb) Additional file 4: Figure S1. Interaction networks for the interaction partners of mASAL generated using STRING database. Homologs of identified *R. solani* interactors for mASAL were taken either from *Saccharomyces cerevisiae* (Actin, ATPase, and 14-3-3 protein) or from human (HSP70) and analysed in STRING database. In each individual case nodes represent different proteins in the network and edges represent functional links between them. Colours of the edges represent the type of evidence available for the said interactions. Green represents neighbourhood, red represents gene fusion, blue represnts cooccurence, violet represents coexpression, purple represents experiments, cyan represents databases, olive green represents text mining, light violet represents homology.(DOCX 209 kb) Additional file 5: Table S2. Predicted functional partners of the cellular candidate interactors of mASAL. (DOC 55 kb)

## Abbreviations

ASAL: *Allium sativum* Leaf Agglutinin
MMP: Mitochondrial membrane potential
ROS: Reactive oxygen species
PCD: Programmed cell death
PBS: Phosphate buffered saline
2-D PAGE: Two-dimensional polyacrylamide gel electrophoresis
IEF: Isoelectric focusing
