# Supplementary material for: Deciphering the mode of action of a mutant Allium sativum Leaf Agglutinin (mASAL), a potent antifungal protein on Rhizoctonia solani
Source: BMC Microbiol. 2015 Oct 26;15:237. doi: 10.1186/s12866-015-0549-7 (PMC4623900; doi:10.1186/s12866-015-0549-7)
Supplement: Additional file 1: Figure S1. — Nuclei counts in hyphae of control and mASAL treated fungal hyphae. Hyphae were harvested and stained with DAPI. The number of nuclei between the hyphal tip and the first two septum was counted manually. For the control type and mASAL treated sample, the graph shows the average number ± S.D. of nuclei counted in at three different time points (*, P < 0.05). (DOCX 636 kb) [file 12866_2015_549_MOESM1_ESM.docx]

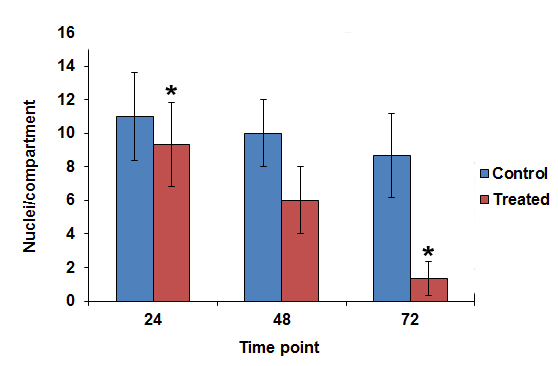


**Fig S1 Nuclei counts in hyphae of control and mASAL treated fungal hyphae.** Hyphae were harvested and stained with DAPI. The number of nuclei between the hyphal tip and the first two septum was counted manually. For the control type and mASAL treated sample, the graph shows the average number ± S.D. of nuclei counted in at three different time points (*, P<0.05).
