# Supplementary material for: Deciphering the mode of action of a mutant Allium sativum Leaf Agglutinin (mASAL), a potent antifungal protein on Rhizoctonia solani
Source: BMC Microbiol. 2015 Oct 26;15:237. doi: 10.1186/s12866-015-0549-7 (PMC4623900; doi:10.1186/s12866-015-0549-7)
Supplement: Additional file 3: — LC MS/MS analysis of identified interactors of mASAL from R.solani Matched peptides highlighted in yellow. Green colour indicated probable sites of mutation and modification. (DOCX 2225 kb) [file 12866_2015_549_MOESM3_ESM.docx]

**Identification of receptor proteins from *R.solani* through LC MS/MS analyses**

**
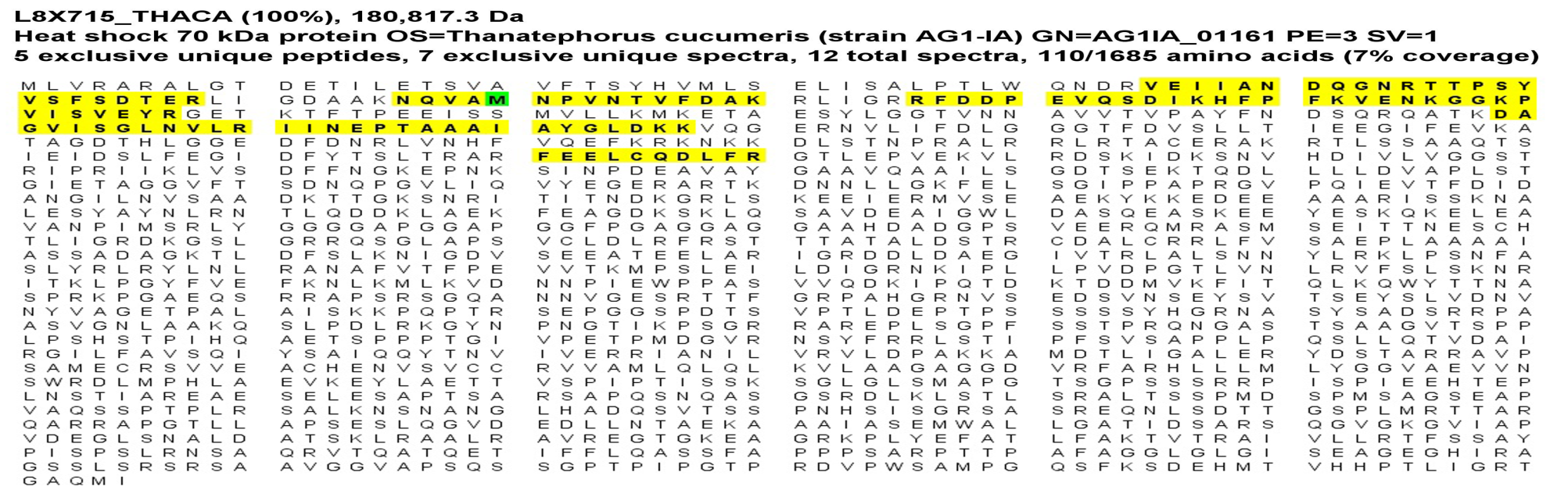
Spot 1**

**Peptide Match Summary:**

| Matched peptide seq | **Probability** | **Mascot ion score** | **Observed**  **mass** | **Actual**  **mass** | **Charge** | **Delta**  **(da)** | **Delta**  **(PPM)** | **Start** | **Stop** | **Modifications** |
| --- | --- | --- | --- | --- | --- | --- | --- | --- | --- | --- |
| (R )VEIIANDQGNR(T) | **100%** | **84.0** | **614.82** | **1227.62** | **2** | **-0.00074** | **-0.60** | **45** | **55** |  |
| (R )TTPSYVSFSDTER(L) | **100%** | **73.9** | **745.34** | **1488.67** | **2** | **0.00016** | **0.11** | **56** | **68** |  |
| (K)NQVAMNPVNTVFDAK(R ) | **97%** | **51.2** | **832.41** | **1662.81** | **2** | **0.0015** | **0.89** | **76** | **90** | **Oxidation (+16)** |
| (R )RFDDPEVQSDIK(H) | **99%** | **56.5** | **483.57** | **14447.69** | **2** | **-0.0013** | **-0.88** | **96** | **107** |  |
| (K)HFPFKVENK(G) | **72%** | **40.0** | **382.54** | **1144.60** | **3** | **0.00054** | **0.47** | **108** | **116** |  |
| (K)GGKPVISVEYR(G) | **97%** | **49.6** | **402.23** | **1203.66** | **3** | **0.00093** | **0.77** | **117** | **127** |  |
| (K)DAGVISGLNVLR(I) | **94%** | **46.4** | **607.35** | **1212.68** | **2** | **-0.00044** | **-0.36** | **179** | **190** |  |
| (R )IINEPTAAAIAYGLDKK(V) | **100%** | **76.8** | **596.67** | **1786.98** | **3** | **0.00026** | **0.15** | **191** | **207** |  |
| (R )FEELCQDLFR(G) | **68%** | **34.5** | **678.82** | **1355.62** | **2** | **0.00035** | **0.26** | **321** | **330** | **Carbamidomethyl (+57)** |

**
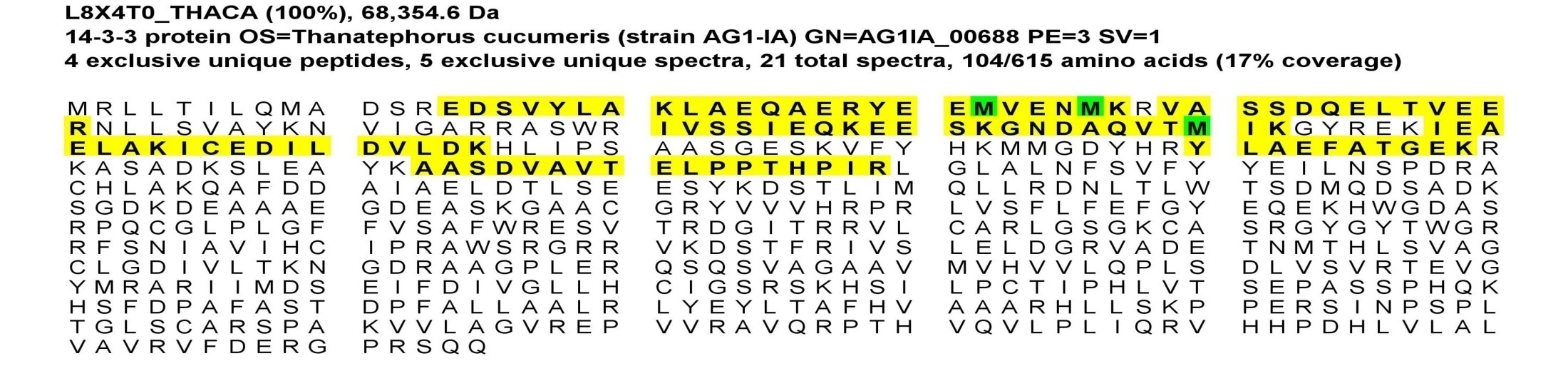
Spot 2**

**Peptide Match Summary:**

| Matched peptide seq | **Probability** | **Mascot ion score** | **Observed**  **mass** | **Actual**  **mass** | **Charge** | **Delta**  **(da)** | **Delta**  **(PPM)** | **Start** | **Stop** | **Modifications** |
| --- | --- | --- | --- | --- | --- | --- | --- | --- | --- | --- |
| (R)EDSVYLAK(L) | **87%** | **50.2** | **467.74** | **923.46** | **2** | **0.00082** | **0.88** | **14** | **21** |  |
| (K) LAEQAERYEE**M**VENMK( R) | **99%** | **46.8** | **668.30** | **2001.88** | **3** | **1.0** | **-0.098** | **22** | **37** | **Oxidation(+16)** |
| (R) VASSDQEITVEER(N) | **100%** | **101.9** | **731.85** | **1461.69** | **2** | **-0.0017** | **-1.2** | **39** | **51** |  |
| (R )IVSSIEQKEESK(G) | **99%** | **69.8** | **688.87** | **1375.72** | **2** | **0.00060** | **0.43** | **71** | **82** |  |
| (K)GNDAQVT**M**IK(G) | **100%** | **76.3** | **546.77** | **1091.53** | **2** | **0.00026** | **0.24** | **83** | **92** | **Oxidation(+16)** |
| (K)IEAELAK(I) | **90%** | **63.3** | **387.22** | **772.43** | **2** | **-0.00036** | **-0.47** | **98** | **104** | **Carbamidomethyl (+57)** |
| (K)ICEDILDVLDK(H) | **100%** | **102.6** | **666.84** | **1331.66** | **2** | **-0.00091** | **-0.53** | **105** | **115** |  |
| (R)YLAEFATGEK(R) | **100%** | **72.1** | **564.78** | **1127.55** | **2** | **0.000062** | **-0.055** | **140** | **149** |  |
| (K)AASDVAVTELPPTHPIR(L) | **100%** | **74.2** | **887.48** | **1772.94** | **2** | **0.0012** | **0.70** | **163** | **179** |  |

**
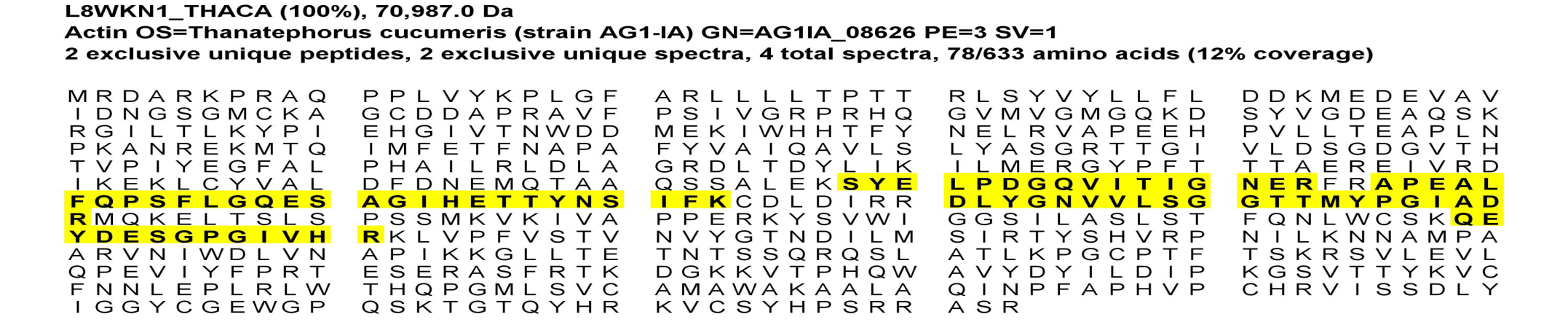
Spot 3**

**Peptide Match Summary:**

| Matched peptide seq | **Probability** | **Mascot ion score** | **Observed**  **mass** | **Actual**  **mass** | **Charge** | **Delta**  **(da)** | **Delta**  **(PPM)** | **Start** | **Stop** | **Modifications** |
| --- | --- | --- | --- | --- | --- | --- | --- | --- | --- | --- |
| (R)AVFPSIVGRPR(H) | **96%** | **53.3** | **599.86** | **1197.70** | **2** | **0.000078** | **0.065** | **68** | **78** |  |
| (R)HQGV**M**VGMGQK(D) | **100%** | **77.0** | **594.29** | **1186.56** | **2** | **-0.00018** | **-0.15** | **79** | **89** | **Oxidation(+16)** |
| (K)DSYVGDEAQSK(R) | **100%** | **86.2** | **599.76** | **1197.52** | **2** | **0.00016** | **0.13** | **90** | **100** |  |
| (K)YPIEHGIVTNWOD**M**EK(I) | **99%** | **42.0** | **654.97** | **1961.88** | **3** | **0.00043** | **0.22** | **108** | **123** | **Oxidation(+16)** |
| (K)IWHHTFYNELR(V) | **100%** | **61.5** | **505.92** | **1514.74** | **3** | **-0.00094** | **-0.00094** | **124** | **134** |  |
| (R)VAPEEHPVLLTEAPLNPK(A) | **100%** | **73.6** | **977.54** | **1953.06** | **2** | **0.00062** | **0.00062** | **135** | **152** |  |
| (K)SYELPDGQVITIGNER(F) | **95%** | **53.7** | **895.95** | **1789.88** | **2** | **-0.00010** | **-0.057** | **278** | **293** |  |

**
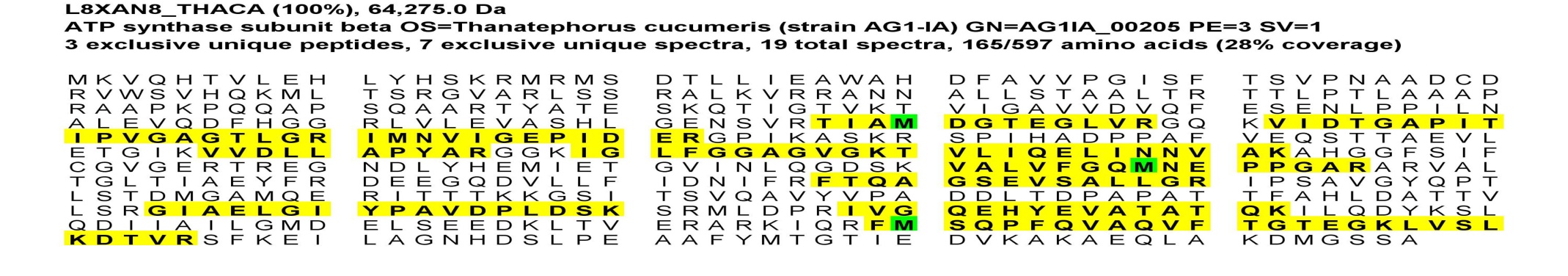
Spot 4**

**Peptide Match Summary:**

| Matched peptide seq | **Probability** | **Mascot ion score** | **Observed**  **mass** | **Actual**  **mass** | **Charge** | **Delta**  **(da)** | **Delta**  **(PPM)** | **Start** | **Stop** | **Modifications** |
| --- | --- | --- | --- | --- | --- | --- | --- | --- | --- | --- |
| (R) TIA**M**DGTEGLVR (G) | **100%** | **73.8** | **639.82** | **1277.63** | **2** | **-0.00074** | **-0.58** | **177** | **188** | **Oxidation (+16)** |
| (K)VIDTGAPITIPVGAGTLGR(I) | **100%** | **74.3** | **904.52** | **1807.02** | **2** | **0.00026** | **0.14** | **192** | **210** |  |
| (R)IMNVIGEPIDER(G) | **99%** | **64.6** | **693.36** | **1384.70** | **2** | **0.00018** | **0.13** | **211** | **222** |  |
| (K)VVDLLAPYAR(G) | **95%** | **52.1** | **558.82** | **1115.63** | **2** | **0.00078** | **0.70** | **256** | **265** |  |
| (K)IGLFGGAGVGK(T) | **98%** | **59.0** | **488.28** | **974.56** | **2** | **-0.000042** | **-0.043** | **269** | **279** |  |
| (K)TVLIQELINNVAK(A) | **100%** | **84.1** | **727.93** | **1453.85** | **2** | **0.00046** | **0.31** | **280** | **292** |  |
| (K)VALVFGQ**M**NEPPGAR(A) | **100%** | **69.9** | **801.41** | **1600.80** | **2** | **0.0012** | **0.75** | **331** | **345** | **Oxidation (+16)** |
| (R)FTQAGSEVSALLGR(I) | **100%** | **99.2** | **718.38** | **1434.75** | **2** | **0.00100** | **0.70** | **377** | **390** |  |
| ( R) GIAELGIYPAVDPLDSK(S) | **100%** | **89.1** | **879.47** | **1756.92** | **2** | **-0.0017** | **-0.97** | **454** | **470** |  |
| (R ) IVGQEHYEVATATQK(I) | **100%** | **114.2** | **837.43** | **1672.84** | **2** | **0.0024** | **1.4** | **478** | **492** |  |
| (R )FMSQPFQVAQVFTGTEGK(L**)** | **100%** | **134.2** | **1009.49** | **2016.96** | **2** | **0.00016** | **0.081** | **529** | **546** | **Oxidation (+16)** |
| (K)LVSLKDTVR(S) | **97%** | **57.1** | **515.82** | **1029.62** | **2** | **0.00096** | **0.93** | **547** | **555** |  |
