# Supplementary material for: Deciphering the mode of action of a mutant Allium sativum Leaf Agglutinin (mASAL), a potent antifungal protein on Rhizoctonia solani
Source: BMC Microbiol. 2015 Oct 26;15:237. doi: 10.1186/s12866-015-0549-7 (PMC4623900; doi:10.1186/s12866-015-0549-7)
Supplement: Additional file 4: Figure S1. — Interaction networks for the interaction partners of mASAL generated using STRING database. Homologs of identified R. solani interactors for mASAL were taken either from Saccharomyces cerevisiae (Actin, ATPase, and 14-3-3 protein) or from human (HSP70) and analysed in STRING database. In each individual case nodes represent different proteins in the network and edges represent functional links between them. Colours of the edges represent the type of evidence available for the said interactions. Green represents neighbourhood, red represents gene fusion, blue represnts cooccurence, violet represents coexpression, purple represents experiments, cyan represents databases, olive green represents text mining, light violet represents homology.(DOCX 209 kb) [file 12866_2015_549_MOESM4_ESM.docx]

**
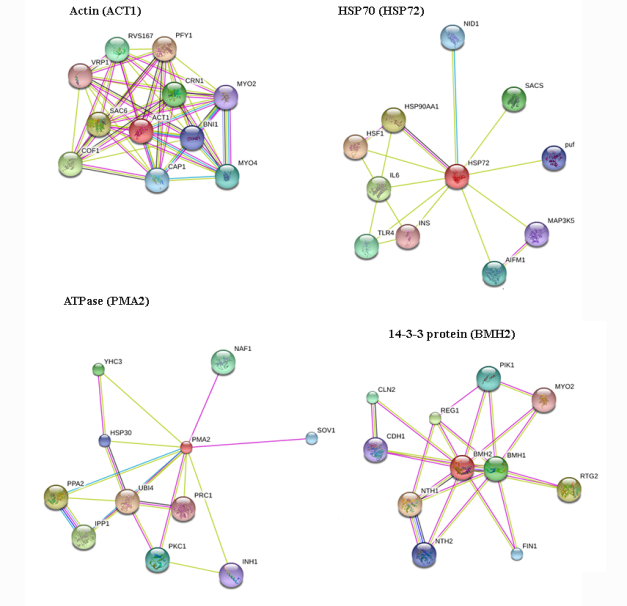
**

**Figure S1. Protein interaction networks for the interaction partners of mASAL generated using STRING database.** Homologues of identified *R. solani* interactors for mASAL were taken either from *Saccharomyces cerevisiae* (Actin, ATPase, ABC transporter and 14-3-3 protein) or from human (HSP70) and analysed in STRING database. In each individual case nodes represent different proteins in the network and edges represent functional links between them. Colours of the edges represent the type of evidence available for the said interactions. Green represents neighbourhood, red represents gene fusion, blue represents cooccurence, violet represents coexpression, purple represents experiments, cyan represents databases, olive green represents text mining, light violet represents homology.

**Table S1.** Predicted functional partners of the cellular candidate interactors of mASAL.

| mASAL interactor | Homologues analysed in STRING database | Predicted functional partners | Major cellular functions affected |
| --- | --- | --- | --- |
| Actin | ACT1 (*Saccharomyces cerevisiae*) | PFY1 (Profilin), | Cytoskeletal structural reorganisation, development and maintenance of cell polarity, formation of endocytic vesicles and budding site selection, cellular growth. |
|  |  | SAC6 (Fimbrin) |  |
|  |  | COF1 (Cofilin) |  |
|  |  | CRN1 (Coronin like protein) |  |
|  |  | RVS167 (reduced viability upon starvation protein) |  |
|  |  | MYO4 (Myosin 4) |  |
|  |  | CAP1 (F-actin capping protein subunit alpha) |  |
|  |  | BNI1 |  |
|  |  | MYO2 (Myosin 2) |  |
|  |  | VPR1 (Verprolin) |  |
| HSP70 | HSP72 (*Homo sapiens*) | HSF1 (Heat shock transcription factor 1) | Cellular growth, differentiation and programmed cell death. |
|  |  | HSP90AA1 (Heat shock protein 90 kDa alpha) |  |
|  |  | IL6 (Interleukin 6) |  |
|  |  | SACS (sacsin) |  |
|  |  | TLR4 (toll like Receptor 4) |  |
|  |  | AIFM1 (Apoptosis inducing factor, mitochondrion associated 1) |  |
|  |  | NID1 (nidogen 1) |  |
|  |  | puf (NME1-NME2) readthrough |  |
|  |  | MAP3K5 (Mitogen activated protein kinase kinase kinase 5) |  |
|  |  | INS (Insulin) |  |
| ATPase | PMA2 (*Saccharomyces cerevisiae*) | UBI4 (Ubiquitin) | Cellular energy production, vacuolar transport, cellular growth, protein translocation across membranes, maintaining protein homeostasis. |
|  |  | PPA2 (Inorganic pyrophosphatase) |  |
|  |  | IPP1 (Inorganic pyrophosphatase) |  |
|  |  | YHC3 (protein BTN1 precursor) |  |
|  |  | NAF1 |  |
|  |  |  |  |
|  |  | PKC1 (Protein kinase C-like 1) |  |
|  |  | SOV1 |  |
|  |  | HSP30 |  |
|  |  | INH1 (ATPase inhibitor) |  |
|  |  | PRC1 (Carboxypeptidase Y precursor) |  |
| 14-3-3 protein | BMH2 (*Saccharomyces cerevisiae*) | NTH1 (Neutral trehalase) | Regulation of cellular growth, cell cycle and programmed cell death. |
|  |  | RTG2 (Retrograde regulation protein 2) |  |
|  |  | REG1 (Protein Hex2) |  |
|  |  | BMH1 |  |
|  |  | CLN2 (G1/S specific cyclin) |  |
|  |  | PIK1 (Phosphatidyl inositol kinase 1) |  |
|  |  | FIN1 (Filament protein) |  |
|  |  | NTH2 (Probablee trehalase) |  |
|  |  | CDH1 (APC/C activator protein) |  |
|  |  | MYO2 (Myosin-2) |  |
